# Pathogenicity Characterization of Prevalent-Type *Streptococcus dysgalactiae* subsp. *equisimilis* Strains

**DOI:** 10.3389/fmicb.2020.00097

**Published:** 2020-02-04

**Authors:** Miki Matsue, Kohei Ogura, Hironori Sugiyama, Tohru Miyoshi-Akiyama, Yukiko Takemori-Sakai, Yasunori Iwata, Takashi Wada, Shigefumi Okamoto

**Affiliations:** ^1^Department of Clinical Laboratory Science, Faculty of Health Sciences, Institute of Medical, Pharmaceutical and Health Sciences, Kanazawa University, Kanazawa, Japan; ^2^Advanced Health Care Science Research Unit, Institute for Frontier Science Initiative, Kanazawa University, Kanazawa, Japan; ^3^Division of Instrumental Analysis, Engineering and Technology Department, Kanazawa University, Kanazawa, Japan; ^4^Pathogenic Microbe Laboratory, Research Institute, National Center for Global Health and Medicine, Shinjuku, Japan; ^5^Division of Clinical Laboratory Medicine, Kanazawa University, Kanazawa, Japan; ^6^Division of Infection Control, Kanazawa University, Kanazawa, Japan; ^7^Department of Nephrology and Laboratory Medicine, Kanazawa University, Kanazawa, Japan

**Keywords:** *Streptococcus dysgalactiae* subsp. *equisimilis*, clinical isolates, *emm* typing, whole-genome analysis, virulence factors, bacterial growth, skin infection

## Abstract

*Streptococcus dysgalactiae* subsp. *equisimilis* (SDSE) is an emerging human pathogen that causes severe invasive streptococcal diseases. Recent reports have shown that SDSE exhibits high pathogenicity with different mechanisms from that of *Streptococcus pyogenes*, although the two streptococci possess some common virulence factors such as streptolysin, streptokinase, and cell-binding proteins. To date, only a few studies have examined the variety of mechanisms expressing the pathogenicity of SDSE. Among nine SDSE clinical isolates sequenced in this study, we present *in vitro* and *in vivo* analyses of KNZ01 and KNZ03, whose *emm* and multilocus species types (MLSTs) are prevalent in Japan and other countries. For the comparison of pathogenicity, we also utilized the ATCC 12394 strain. The whole-genome analysis showed that KNZ03 and ATCC 12394 are categorized into an identical clonal complex by MLST and are phylogenetically close. However, the three strains exhibited different characteristics for pathogenicity *in vitro*; ATCC 12394 showed significant cytotoxicity to human keratinocytes and release of streptolysin O (SLO) compared to KNZ01 and KNZ03; KNZ03 exhibited significantly high hemolytic activity, but did not secrete SLO. KNZ01 and KNZ03 adhered to human keratinocytes at a higher rate than ATCC 12394; KNZ03 showed a higher rate of survival after a brief (30 min) incubation with human neutrophils compared to the other two strains; also, KNZ01 grew more rapidly in the presence of human serum. *In vivo* subcutaneous infection commonly resulted in ulcer formation in the three strains 7 days after infection. KNZ01-infected mice showed significant body weight loss 2 days after infection. Besides, on post-infection day 2, only KNZ01 remained in the cutaneous tissues of mice. Scanning electron microscopy analysis revealed that KNZ01 formed an extracellular structure (biofilm), which was probably composed of cell wall-anchoring proteins, in the presence of glucose and human serum. The extracellular structure of ATCC 12394 was also changed dramatically in response to culture conditions, whereas that of KNZ03 did not. Our study proposed that each SDSE strain possesses different virulence factors characteristics for mediating pathogenicity in humans.

## Introduction

*Streptococcus dysgalactiae* subsp. *equisimilis* (SDSE) belongs to β-hemolytic streptococci and possesses Lancefield group C or G antigens (rarely, A antigen; Takahashi et al., 2011). SDSE is recognized as a common colonizer of the pharynx, skin, gastrointestinal tract, and female genital tract (Yung et al., 2019). Although SDSE is a part of the normal human flora, SDSE causes a diversity of diseases ranging from wound infection, erysipelas, and cellulitis to life-threatening necrotizing fasciitis and streptococcal toxic shock syndrome (Rantala, 2014). Moreover, some studies have reported that SDSE is associated with pharyngitis, acute post-streptococcal glomerulonephritis, and acute rheumatic fever (Haidan et al., 2000; Bassett et al., 2009). These clinical features of diseases caused by SDSE closely resemble *Streptococcus pyogenes* (GAS) infections (Brandt and Spellerberg, 2009).

Similar to GAS, SDSE possesses various virulence factors including M protein, streptolysin O (SLO), streptolysin S (SLS), streptokinase, hyaluronidase, C5a peptidase, and others (Takahashi et al., 2011). In contrast, SDSE lacks several key virulence factors present in GAS, such as superantigens other than *speG*, the cysteine protease *speB*, the hyaluronic synthesis operon *hasA* and *hasB* (Watanabe et al., 2016), and an inhibitor of complement activation *sic* (Wajima et al., 2016).

The number of reports on the pathogenicity of SDSE is increasing. Most patients with invasive SDSE infections are elderly people with underlying diseases such as diabetes mellitus. The mean age of adult patients with invasive SDSE infection was older than those infected with GAS (Cohen-Poradosu et al., 2004; Wajima et al., 2016). Our group reported that an SDSE strain causes severe pathogenicity to diabetic mice compared with GAS strains (Ogura et al., 2018). Genteluci et al. (2015) reported that 59.3% of the 118 SDSE clinical isolates were able to form biofilm *in vitro*, and all of the strains formed biofilm in mice. They also found that the capacity to form biofilm *in vitro* of even weak biofilm-forming strains increased after animal passage. Although SDSE has been considered as less virulent streptococci than GAS, these recent reports showed high pathogenicity of SDSE infection with various clinical features.

For invasive cases of GAS infection, *emm1* commonly represented the dominant type in Europe, North America, and Japan (Wajima et al., 2008; Gherardi et al., 2018). The increase in virulence of the *emm1* GAS strain can be attributed to its diversification through phage mobilization and the ability to sense and adapt to different host environments (Aziz and Kotb, 2008). Plainvert et al. (2012) reported that the dominance of the *emm1* type was associated with both the number of necrotizing fasciitis cases and the distribution of phage-associated superantigen genes. However, predominant *emm* types of SDSE varied per country. For example, in central Taiwan, the *stG*10, *stG*245, *stG*840, *stG*6.1, and *stG*652 strains predominated (Ma et al., 2017), whereas *stG*485 and *stG*6.1 were predominant in Jerusalem and Austria, respectively (Cohen-Poradosu et al., 2004; Leitner et al., 2015). In Japan, *stG*6792 was the most prevalent, followed by *stG*485 and *stG*245 (Wajima et al., 2016; Ikebe et al., 2018). Besides *emm* typing, SDSE is categorized by multilocus sequence typing (MLST) (McMillan et al., 2010). The MLST scheme showed that most SDSE *emm* types are found in multiple sequence types (STs) and that, the same ST can harbor different *emm* types. To date, only a few studies have examined the relationship between the diverse virulence factors unique to each SDSE strain and *emm*/MLS typings. Here, we examined the nine Lancefield group G SDSE clinical strains isolated from the Kanazawa University Hospital in Japan from 2014 to 2016. To reveal the mechanisms of each SDSE and thus, express the pathogenicity, we chose one each of an *stG*6792-ST17 (Clonal complex 17, CC17) strain KNZ01 and *stG*245-ST127 (CC25) strain KNZ03, the predominant *emm* types in Japan, and examined the genomic features, activities of SLS and SLO, adherence and cytotoxicity to human keratinocytes, anti-phagocytic activity, growth activity in human serum, biofilm formation, and pathogenicity after subcutaneous infection in mice. We utilized *stG*166-ST25 (CC25) Lancefield group G SDSE strain ATCC 12394, whose transfer is available, for pathogenicity comparisons.

## Materials and Methods

### Bacterial Strains and Culture Conditions

Nine Lancefield group G SDSE strains were isolated from the Kanazawa University Hospital (Table 1): KNZ01 was isolated from the joint fluid; six strains (KNZ03, KNZ04, KNZ06, KNZ07, KNZ12, and KNZ15) were isolated from patients with bacteremia via blood cultures; KNZ10 and KNZ16 via swabbing open wounds. After Lancefield antigen and hemolysis tests on blood agar plates, the β-hemolytic group G strains were identified as SDSE using the API 20 Strep kit (bio Merieux). Group G SDSE strain ATCC 12394 was obtained from the American Type Culture Collection (ATCC)^1^. SDSE strains KNZ01, KNZ03, and ATCC 12394 were cultured overnight in THY medium consisting of Todd-Hewitt broth (Becton Dickinson, Franklin Lakes, NJ, United States) and 0.2% yeast extract (Becton Dickinson) at 37°C under 5% CO_2_ atmosphere. Before use in experiments, bacterial cells were cultured overnight, suspended in fresh THY medium, and incubated until the optical density at a wavelength of 600 nm (OD_600_) reached from 0.01 to 0.4–0.5. All experiments were conducted in accordance with the WHO Laboratory biosafety manual and the institutional safety procedure of Kanazawa University.

**TABLE 1:** *Streptococcus dysgalactiae* subsp. *equisimilis* strains isolated from Kanazawa University Hospital from 2014 to 2016.

	Clinical Specimen	Genome size (bp)	Number of CDSs	*emm* type	Sequence type (MLST)	Clonal complex	Allele number (MLST)						
							
Strains							*gki*	*gtr*	*murl*	*mutS*	*recP*	*xpt*	*atoB*
KNZ01	Joint fluid	2,077,921	1,975	*stG6792*	17	17	4	4	1	2	17	6	2
KNZ03	Blood	2,155,317	2,069	*stG245*	127	25	3	2	1	5	7	33	3
KNZ04	Blood	2,163,978	2,031	*stG245*	127	25	3	2	1	5	7	33	3
KNZ06	Blood	2,109,503	1,959	*stG480*	151	15	3	3	2	2	28	8	2
KNZ07	Blood	2,045,272	1,919	*stG6792*	17	17	4	4	1	2	17	6	2
KNZ10	Open pus	2,055,511	1,929	*stG245*	17	17	4	4	1	2	17	6	2
KNZ12	Blood	2,093,560	1,941	*stG6792*	520	17	4	10	1	2	17	6	2
KNZ15	Blood	2,091,136	1,978	*stG653*	17	17	4	4	1	2	17	6	2
KNZ16	Open pus	2,164,479	2,072	*stG166*	148	25	3	2	1	17	7	4	3
ATCC 12394	–	2,159,491	2,146	*stG166*	25	25	3	2	1	5	7	4	3

### Cell Line and Culture Conditions

The human keratinocyte cell line (HaCaT cells; Boukamp et al., 1988) was cultured in Dulbecco’s modified Eagle’s medium (DMEM; FUJIFILM Wako Pure Chemical Corporation, Osaka, Japan) containing 8% fetal bovine serum (FBS; Biosera, Kansas, MO, United States) and 100 U/ml penicillin/100 μg/ml streptomycin/250 ng/ml amphotericin B (FUJIFILM Wako Pure Chemical Corporation) at 37°C under 5% CO_2_ atmosphere.

### Whole-Genome Analysis

KNZ01 and KNZ03 strains were sequenced using an MiSeq system. DNA was prepared for Illumina library construction by Nextera XT library kits (Illumina, San Diego, CA, United States) according to the manufacture’s instruction. Approximately 1 million 301 bp pair-end reads were trimmed based on base quality (quality score limit = 0.05) and removed if the reads had more than two ambiguous nucleotides or were less than 15 bp in length followed by *de novo* assembly to construct contigs using the CLC genomics workbench software program (CLC bio, Aarhus, Denmark) with the parameters; mapping mode of simple contig sequences creation (fast), minimum contig length = 500, automatic bubble sizing, automatic word sizing, scaffold performing, and auto-detection of paired distances. The whole genomes have been registered with DNA Data Bank of Japan database under BIOSAMPLE accession numbers SAMD00191542 (KNZ01), SAMD00191543 (KNZ03), SAMD00191544 (KNZ04), SAMD00191545 (KNZ06), SAMD00191546 (KNZ07), SAMD00191547 (KNZ10), SAMD00191548 (KNZ12), SAMD00191549 (KNZ15), SAMD00191550 (KNZ16). CDS sequences were extracted by DFAST (Tanizawa et al., 2018). *emm* type was determined by a BLAST search against the database in the Centers for Disease Control and Prevention^2^. MLST profiles were submitted to pubMLST (Jolley and Maiden, 2010; McMillan et al., 2010). Average nucleotide identity (ANI) was calculated using the ANI Calculator (Yoon et al., 2017). Forty-eight SDSE genome sequences were obtained from the National Center for Biotechnology Information website. Single nucleotide polymorphisms (SNPs) of core genomes were extracted by Parsnp webtool (Treangen et al., 2014) using the SDSE 167 core genome as a reference (Watanabe et al., 2013). A phylogenetic tree was constructed using the MEGA5 software with the neighbor-joining method and 100 bootstraps (Tamura et al., 2011).

### Hemolytic Assay

The hemolytic activity of SDSE strains was measured as described previously (Ogura et al., 2018). SDSE strains were cultured overnight in THY medium supplemented with 1% Tween 80 (Sigma Aldrich, St. Louis, MO, United States), centrifuged at 1500 × *g*, and resuspended in fresh THY medium at OD_600_ = 1.0. Sheep whole blood (5 ml) in Alsever’s solution (Nippon Bio-Test Laboratories, Inc., Saitama, Japan) was centrifuged at 1200 × *g* for 20 min, washed twice with sterile phosphate-buffered saline (PBS), and resuspended in 50 ml PBS containing 2.5% (v/v) bovine serum albumin (FUJIFILM Wako Pure Chemical Corporation). After pre-incubation of the red blood cell (RBC) solution at 37°C for 15 min in the presence or absence of 50 mM 2-mercaptoethanol (2-ME; SLO enhancer; Nakalai Tesque, Kyoto, Japan; Van Epps and Andersen, 1971) and 0.01% trypan blue (TB; SLS inhibitor; FUJIFILM Wako Pure Chemical Corporation; Taketo and Taketo, 1982), bacterial solution and sheep RBC solution were incubated at a ratio of 1:10 at 37°C for 2 h under 5% CO_2_ atmosphere. The sheep RBC solution incubated with 0.2% Triton X-100 and THY medium only were used as hemolysis positive and negative controls, respectively. After centrifugation at 2000 × *g* for 5 min, the absorbance of the supernatants was measured at a wavelength of 550 nm.

### Cytotoxic Assay

*Streptococcus dysgalactiae* subsp. *equisimilis* strains at mid-exponential phase (OD_600_ = 0.4–0.5) were centrifuged, washed with PBS, and resuspended in DMEM. HaCaT cells were seeded at 5 × 10^4^ per well in 96-well plates in DMEM and cultured at 37°C under 5% CO_2_ atmosphere. Confluent HaCaT cells were washed twice with DMEM and inoculated with SDSE strains at a multiplicity of infection (MOI) of 10. HaCaT cells with DMEM only was used as a cytotoxic negative control. After 2 h incubation at 37°C under 5% CO_2_ atmosphere, the supernatants were collected and centrifuged at 1500 × *g* for 10 min. Cytotoxic activity was measured using the CytoTox-Fluor^TM^ Cytotoxicity Assay kit (Promega Corp., Madison, WI, United States) according to the manufacturer’s instructions (Running et al., 1999).

### Western Blot Analysis

*Streptococcus dysgalactiae* subsp. *equisimilis* strains were cultured overnight in THY medium supplemented with 1% Tween 80, centrifuged at 1500 × *g*, and resuspended in fresh THY medium at OD_600_ = 0.1 in the absence or presence of ProteoGuard EDTA-Free Protease Inhibitor Cocktail (Takara Bio Inc., Shiga, Japan). After incubation at 37°C for 2 h, the supernatants were collected for Western blot analysis. Anti-SLO primary (#64-001) and anti-rabbit HRP-conjugated secondary (#HAF008) antibodies were purchased from Bio Academia (Suita, Osaka, Japan) and R&D Systems (Minneapolis, MN, United States), respectively. HRP-bound protein bands were detected by Immobilon Western Chemiluminescent HRP Substrate (#WBKLS0500; Merck Millipore, Bedford, MA, United States) and visualized by LuminoGraph I (ATTO). Band intensities were measured using ImageJ software (Schneider et al., 2012).

### Adherence and Growth Measurement

*Streptococcus dysgalactiae* subsp. *equisimilis* adherence to HaCaT cells was quantified as described previously (Ozeri et al., 1998; Pappesch et al., 2017). Briefly, SDSE strains at the mid-exponential phase were centrifuged, washed with PBS, and resuspended in DMEM (Fraction A). Confluent HaCaT cells in 24-well plates were washed twice with PBS and inoculated with SDSE strains at an MOI of 10. For HaCaT cells-free control, SDSE strains were suspended in 24-well plates without HaCaT cells (Fraction B). After 2 h incubation at 37°C under 5% CO_2_ atmosphere, the culture supernatants were collected (Fraction C). After washing thrice with PBS, the cell-wash fluids were also collected (Fraction D). To detach the HaCaT cells, trypsin/EDTA (FUJIFILM Wako Pure Chemical Corporation) was added, followed by lysis of HaCaT cells with sterile distilled water (Fraction E). The colony-forming units (CFUs) of Fractions A to E were measured by serial dilution in PBS and plating on THY agar plates. Adherence (%) was defined as follows: [E/(C + D + E)] × 100. Growth (%) was defined as follows: [B or (C + D + E)/A] × 100.

### Assay for Survival in the Presence of Human Neutrophils

Surviving SDSE strains in the presence of human neutrophils were assessed as described previously with some modifications (Pappesch et al., 2017). Human neutrophils were isolated from human whole blood using Polymorphprep (Abbott Diagnostics Technologies AS, Oslo, Norway) according to the manufacturer’s instructions. Fresh human blood was collected from 23- to 36-year-old volunteers. Human neutrophils were suspended in RPMI 1640 (Nissui Pharmaceutical Co., Ltd., Tokyo, Japan). SDSE strains were grown in THY medium overnight and washed with PBS. For opsonization, 1 × 10^6^ to 2 × 10^6^ CFU/ml were incubated for 30 min with 10% human serum at room temperature. Opsonized SDSE strains were incubated with 1 × 10^6^ human neutrophils/ml at an MOI of 1–2 at 37°C for 30 min or 2 h under 5% CO_2_ atmosphere. After lysis of neutrophils by sterile diluted water, CFUs were measured. CFUs of SDSE strains before incubation with human neutrophils were used as input controls.

### Growth Assay in Human Serum

*Streptococcus dysgalactiae* subsp. *equisimilis* strains were incubated in RPMI 1640 with 5% fresh human serum at 37°C for 30 min or 2 h under 5% CO_2_ atmosphere. CFUs were measured by serial dilution in PBS and plating on THY agar plates.

### Subcutaneous Infection

Subcutaneous infection was performed as described previously (Gera et al., 2014). SDSE strains were grown in THY medium to mid-exponential phase (OD_600_ = 0.5), washed, and resuspended in PBS. Mice were anesthetized by intraperitoneal administration of the mixture of Domitor (0.3 mg/kg; Nippon Zenyaku Kogyo Co., Ltd., Fukushima, Japan), Dormicum (4 mg/kg; Astellas Pharma, Inc., Tokyo, Japan), and Vetorphale (5 mg/kg; Meiji Seika Pharma Co., Ltd., Tokyo, Japan). For subcutaneous infection, 2 cm^2^ areas of the back of 6- to 8-week-old female BALB/c mice (Sankyo Labo Service Corp., Inc., Tokyo, Japan) were shaved followed by subcutaneous injection of 100 μl of air to form air pouch and 200 μl of approximately 3.5 × 10^9^ CFU/ml as verified by plating on THY agar plates. After infection, body weight and severity of cutaneous lesions were monitored every day for 7 days.

### Histologic Analysis

For histologic analysis, mice were euthanized by Sevofrane (Maruishi Pharmaceutical Co., Ltd., Osaka, Japan) at 2 days after subcutaneous infection. Cutaneous tissues were fixed with 10% neutral-buffered formalin (FUJIFILM Wako Pure Chemical Corporation). Tissues were stained by Gram Stain Kit (Modified Brown & Brenn; ScyTek Laboratories, Logan, UT, United States) and hematoxylin and eosin (H&E; Muto Pure Chemicals Co., Ltd., Tokyo, Japan) according to the manufacturer’s instructions. Stained tissue sections were examined with an optical microscope (Eclipse E600 with U-III Film Camera System; Nikon Corp., Tokyo, Japan). Images were captured using the imaging software NIS-Elements (Nikon).

### Scanning Electron Microscopy (SEM)

Scanning Electron Microscopy analyses were performed as described previously with some modifications (Honda-Ogawa et al., 2017). SDSE strains were grown overnight in THY medium or RPMI 1640 supplemented with 10% fresh human serum at 37°C under 5% CO_2_ atmosphere. Bacterial cells were fixed with 2.5% glutaraldehyde (FUJIFILM Wako Pure Chemical Corporation) on aluminum plates coated with Matrigel (Corning, Inc., NY, United States) at room temperature for 1 h and washed twice with PBS. After serial dehydration with 50, 80, and 100% ethanol, the samples were soaked in 100% *t*-butyl alcohol (FUJIFILM Wako Pure Chemical Corporation), freeze-dried (Freeze Dryer ES-2030, Hitachi Technologies, Tokyo Japan), and coated with 60% Au-Pd alloy (Hitachi E-1030, Hitachi Technologies; sputtering conditions: 15 mA, 7 Pa, 30 s). The SEM images were obtained using JSM-7100F (JEOL Ltd., Tokyo, Japan).

### Ethics Statement

All animal experiments were carried out in accordance with the Declaration of Helsinki. The protocols of the clinical study, the animal experiment, and the human blood experiment were approved by the Ethical Committee of Kanazawa University. In line with institutional guidelines following local legislation, consent was not required. Clinical isolates were isolated, stored, and utilized for studies on according to the guidelines that are open at the homepage of Kanazawa University Hospital. The opportunity for patients to opt-out was always available. None of the patients were under the age of 16. All the genetic recombinant experiments were conducted in accordance with Law Concerning the Conservation and Sustainable Use of Biological Diversity through Regulations on the Use of Living Modified Organisms and approved by The Kanazawa University Safety Committee for genetic recombinant experiments.

## Results

### Genomic Features of SDSE Strains

**FIGURE 1:**
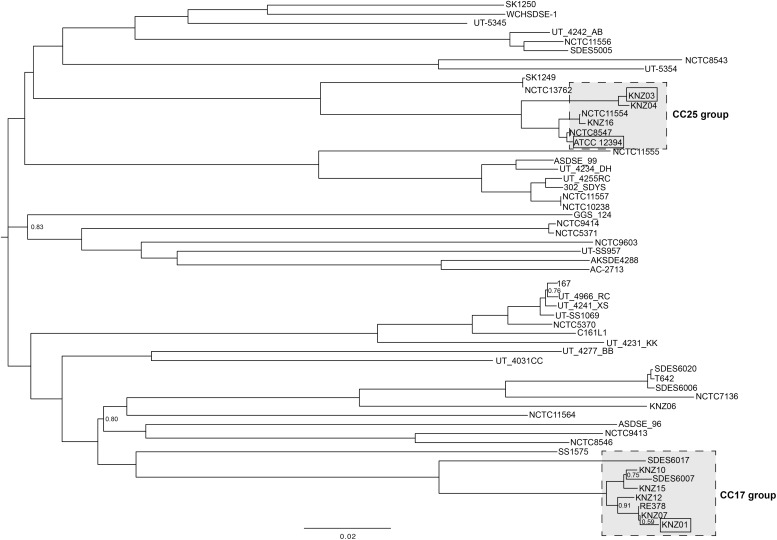
Phylogenetic analysis of SDSE strains using 46,146 SNPs. The phylogenetic tree visualization was conducted using FigTree (software V1.4.1.). Branches without numbers indicate a support value of 1.0.

The genomic features of the nine SDSE strains, including clinical specimen, *emm* type, sequence type (ST), and clonal complex (CC), are shown in Table 1; the most prevalent *emm* types are *stG*6792 and *stG*245, followed by *stG*480, *stG*653, and *stG*166. Five of the nine strains were categorized as CC17 with one novel ST, strain KNZ12; one strain as CC15; the other three strains, as CC25. We chose one *stG*6792/CC17 strain KNZ01 and one *stG*245/CC25 strain KNZ03. Here, we also utilized strain ATCC 12394 (*stG*166/CC25), whose transfer information is available from ATCC, for pathogenicity comparisons; KNZ03 possessed ParC S79YF substitution associated with fluoroquinolone resistance (González et al., 1998). Next, we performed a phylogenetic analysis using concatenated SNPs (Figure 1). The phylogenetic distance between KNZ03 and ATCC 12394 was close; KNZ03 showed 99.7% ANI with ATCC 12394. In contrast, KNZ01 was very close to the other *stG*6792/CC17 SDSE strains, KNZ07 and RE378, with 99.9 and 99.8%, sequence similarity, respectively.

### Hemolytic and Cytotoxic Activity

**FIGURE 2:**
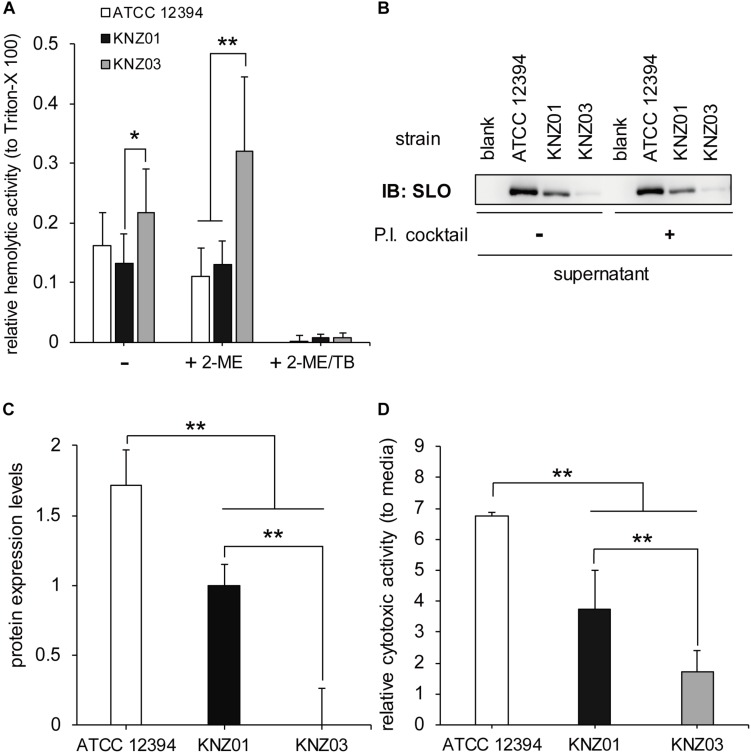
Hemolytic and cytotoxic activities. **(A)** Hemolytic activity. A 5% sheep RBC suspension was added to a bacterial suspension and incubated in the presence or absence of 50 mM 2-ME (activator of SLO) and 0.01% TB (inhibitor of SLS) at 37°C for 2 h. After centrifugation, supernatants were collected, and the absorbance was measured at 540 nm. Data represent the mean ± standard deviation of three separate triplicate experiments. **(B)** Immunoblot with an anti-SLO antibody. The three strains were incubated at 37°C for 2 h in THY medium in the absence or presence of protease inhibitor (P.I.) cocktail. After centrifugation, the culture supernatants were collected for Western blot analysis. **(C)** Densitometric analysis of the immunoblot. Data represent the mean ± standard deviation of two separate sextuplicate experiments. **(D)** Cytotoxicity to human keratinocyte (HaCaT cells). Confluent HaCaT cells were incubated with bacterial suspension (MOI = 10). After 2 h incubation, culture supernatants were collected, centrifuged, and used for cytotoxicity assay. For negative control of cell death, cells were incubated with DMEM containing 8% featal bovine serum (DMEM/FBS) only. Data are mean ± standard deviation of three separate triplicate experiments. *P < 0.05, **P < 0.01, calculated by one-way analysis of variance (ANOVA) with Tukey–Kramer multiple comparison test.

To examine the hemolytic activities, the three SDSE strains were incubated with 5% sheep RBCs for 2 h (Figure 2). Incubation of KNZ03 with sheep RBCs showed significantly higher hemolysis compared with KNZ01. Treatment with 2-mercaptoethanol (+2-ME), an SLO enhancer, resulted in significant enhancement of hemolysis in KNZ03-treated sheep RBCs, indicating that KNZ03 expressed high SLO activities. However, these hemolytic activities were substantially suppressed by trypan blue (+2-ME/TB), an SLS inhibitor. Western blot analysis showed that SLO was not found in the culture supernatant of KNZ03 (Figure 2B). Also, the protease inhibitor cocktail did not affect the amount of SLO, showing that the absence of SLO was not due to protein degradation. These results suggested that KNZ03 secretes high SLS but not SLO. Western blot analysis also showed that ATCC 12394 released significantly higher SLO protein levels than KNZ01 (Figures 2B,C). SLO is known to exhibit cytotoxic activity to mammalian cells (Bhakdi et al., 1985; Palmer, 2001); thus, we examined the cytotoxicity of the SDSE strains to human keratinocytes (HaCaT cells). As shown in Figure 2D, the incubation of HaCaT cells with ATCC 12394 resulted in significant cell death compared with that of KNZ01 and KNZ03; this is consistent with the amounts of SLO protein (Figure 2C). These results indicated that ATCC 12394 expressed the highest cytotoxic activity to human keratinocytes by producing large amounts of SLO protein.

### Adherence to Human Keratinocytes

**FIGURE 3:**
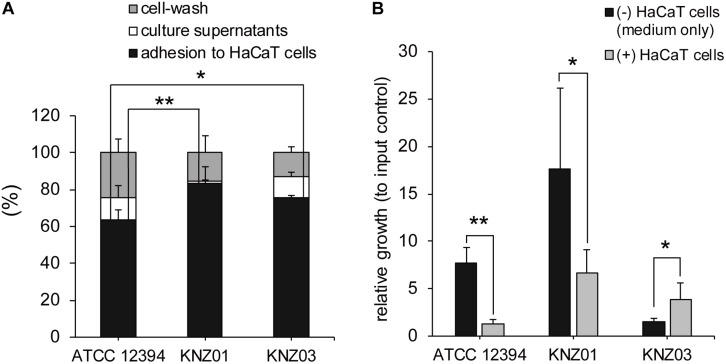
Adherence to human keratinocytes (HaCaT cells) and bacterial growth on HaCaT cells. **(A)** Adherence to HaCaT cells. HaCaT cells were cultured in 24-well plates until 100% confluent and inoculated with bacterial suspension (MOI = 10). After 2 h incubation at 37°C, culture supernatants (white bars) were collected, HaCaT cells were washed thrice with PBS (gray bars), and the cells and adhered bacteria were harvested (black bars). Collected solutions were diluted and plated on THY agar plates to count the CFUs. Data are mean ± standard deviation of two separate triplicate experiments. *P < 0.05, **P < 0.01, calculated by one-way ANOVA with Tukey–Kramer multiple comparison test. **(B)** Bacterial growth in DMEM/FBS in the absence (black bars) or presence (gray bars) of HaCaT cells. After 2 h incubation, culture supernatants and cells were harvested to count the CFUs. Data are mean ± standard deviation of two separate triplicate experiments. *P < 0.05, **P < 0.01, calculated by Welch’s *t*-test.

Adherence and cytotoxicity to keratinocytes are related to severity of skin infections such as necrotizing soft-tissue infections (Humar et al., 2002; Siemens et al., 2015). To investigate adherence of the SDSE strains to human keratinocytes, we counted the number of bacteria attached to HaCaT cells. After 2 h incubation of the SDSE strains with HaCaT cells, the percentage of adherence of ATCC 12394, KNZ01, and KNZ03 was 65, 85, and 75%, respectively (Figure 3A). KNZ01 and KNZ03 showed significantly higher adherence to human keratinocytes than ATCC 12394.

### Bacterial Growth in the Presence of Human Keratinocytes

To examine bacterial growth in the presence of human keratinocytes, the SDSE strains were incubated with DMEM supplemented with 8% inactivated FBS (DMEM/FBS) in the absence or presence of HaCaT cells. We counted the CFUs of inoculated bacteria (input controls), bacteria incubated in the absence of HaCaT cells (DMEM/FBS only) for 2 h, and bacteria incubated in the presence of the cells for 2 h (Figure 3B).

After 2 h incubation in DMEM/FBS only (black bars), CFUs of ATCC 12394 and KNZ01 increased 8 and 18 times, respectively, whereas that of KNZ03 increased only 1.5 times compared with that of input controls. In contrast, in the presence of HaCaT cells (gray bars), ATCC 12394 did not increase. Although KNZ01 increased seven times in the presence of HaCaT cells compared with that of input controls, the growth was significantly lower than that in DMEM/FBS only. Interestingly, contrary to ATCC 12394 and KNZ01, we found that the growth of KNZ03 incubated in the presence of HaCaT cells was significantly 2.6 times higher than that in DMEM/FBS only. These results indicated that the three SDSE strains possessed different mechanisms of growth on host keratinocytes. Although some triggers of KNZ03 in response to attachment to epithetical cell are postulated, no supporting data were obtained. It remains unknown what triggers induce the rapid growth of KNZ03 in the presence of keratinocytes.

### Anti-phagocytic Activity

**FIGURE 4:**
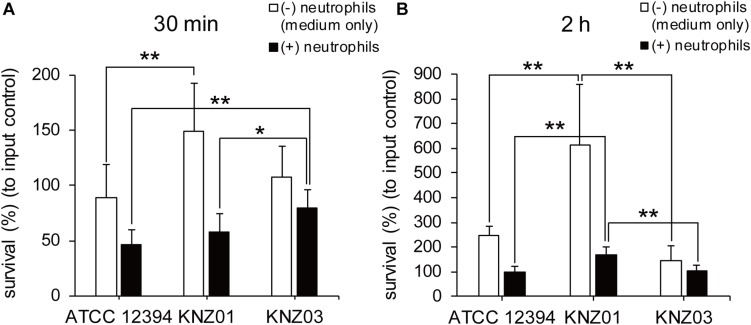
Anti-phagocytic activity and bacterial growth in human serum-containing medium. Survival rates of SDSE strains after incubation with human neutrophils for **(A)** 30 min and **(B)** 2 h. SDSE strains opsonized in human serum for 30 min were incubated in the absence (white bars) or presence (black bars) of human neutrophils (MOI = 1–2) in 10% human serum-containing RPMI 1640 at 37°C. CFUs were measured by serial dilution in PBS and plating on THY agar plates. Data are mean ± standard deviation of three separate triplicate experiments. *P < 0.05, **P < 0.01, calculated by one-way ANOVA with Tukey–Kramer multiple comparison test.

To examine anti-phagocytic activity, the SDSE strains were incubated with human neutrophils in RPMI 1640 containing 10% fresh human serum for 30 min or 2 h followed by counting of CFUs (Figure 4). Compared with CFUs of input controls (100%), CFUs of ATCC 12394, KNZ01, and KNZ03 decreased to 45, 60, and 80%, respectively, after incubation with human neutrophils for 30 min (Figure 4A, black bars). This result indicated that KNZ03 had the highest anti-phagocytic activity against human neutrophils in a short time. After 2 h incubation (Figure 4B, black bars), CFU of KNZ01 increased significantly to 170% compared with that of input control, indicating that KNZ01 showed significantly high growth after avoidance of phagocytosis by human neutrophils. In addition, we found that KNZ01 showed a significant growth in the presence of human serum. As shown in Figure 4B (white bars), after 2 h incubation in human serum without neutrophils, CFU of KNZ01 increased to 600%, whereas those of ATCC 12394 and KNZ03 increased to 250 and 150%, respectively. These results indicated that KNZ01 had the highest growth activity in human serum among the three SDSE strains, which was involved in the highest survival rate of KNZ01 after 2 h incubation with human neutrophils.

### *In vivo* Subcutaneous Infection

**FIGURE 5:**
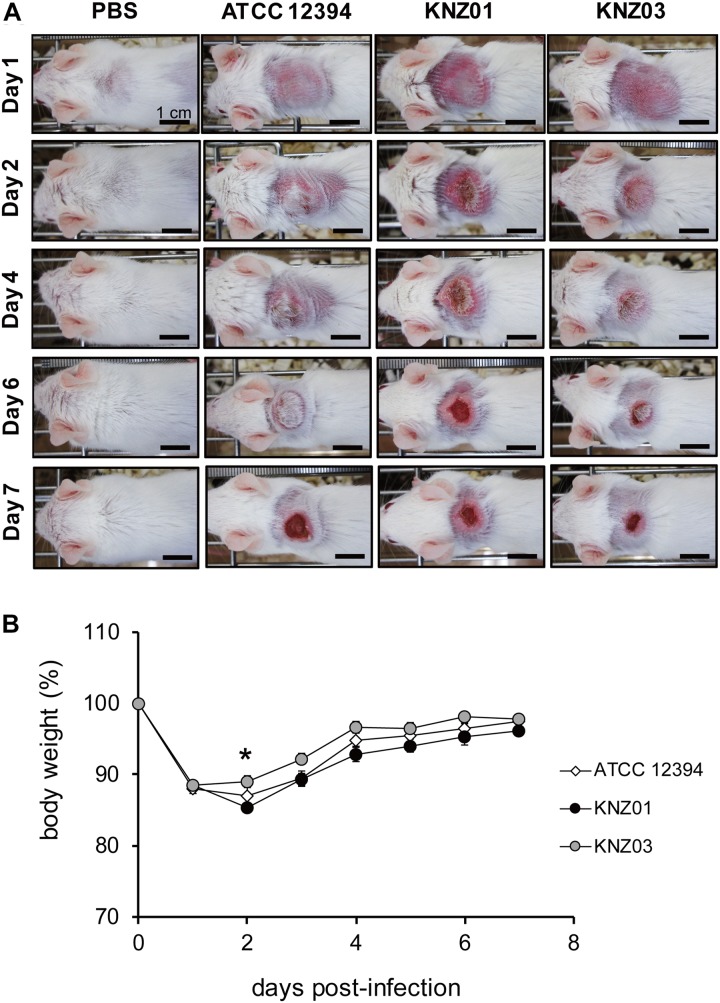
Subcutaneous infection. **(A)** Representative images of mice skin after subcutaneous injection of SDSE strains (*n* = 10/group). Bars indicate 1 cm. **(B)** Changes of body weight after injection. Mice were anesthetized by intraperitoneal administration, and 2 cm^2^ areas of the back were shaved followed by subcutaneous injection of 100 μl of air to form air pouch and 200 μl of approximately 3.5 × 10^9^ CFU/ml. The weight change of KNZ01-infected mice was significant (*P = 0.0137) by one-way ANOVA with Tukey–Kramer multiple comparison test (*n* = 10/group).

**FIGURE 6:**
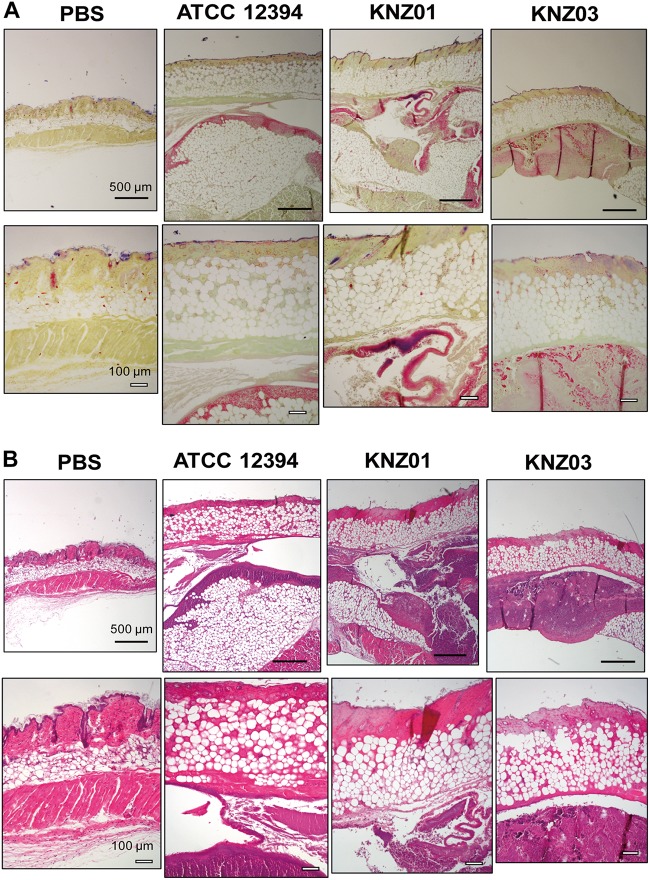
Gram stain and H&E stain images of cutaneous tissues of mice on day 2 after injection of SDSE strains. **(A)** Gram stain and **(B)** H&E images of cutaneous tissues at the infection site on day 2 after injection of SDSE strains. SDSE-infected mice were euthanized by Sevofrane at 2 days after subcutaneous infection. Cutaneous tissues were fixed with 10% neutral-buffered formalin, stained by Gram Stain Kit and hematoxylin and eosin. Stained tissue sections were examined with an optical microscope and images were captured using the imaging software NIS-Elements. Black and white bars indicate 500 and 100 μm, respectively (*n* = 4/group).

To examine the correlation between our *in vitro* observations and the severity of skin infections, we subcutaneously infected the SDSE strains into mice (Figure 5). On day 1 or 2 after infection, mice infected with all strains developed skin lesions with erythema and purulence (Figure 5A). On day 6 or 7 after infection, mice infected with all strains formed ulcerative lesions with epidermal hyperplasia around the sites of infection. These results indicated that ATCC 12394, KNZ01, and KNZ03 showed pathogenicity to mice to cause severe subcutaneous infections with ulcerative lesions. On day 2 after infection, the body weight of mice infected with KNZ01 decreased significantly compared with that of mice infected with KNZ03 (Figure 5B). In addition, erythema and purulence of mice infected with KNZ01 looked most severe among the three SDSE strains, although there was no significant difference in lesion sizes (Figure 5A). Gram stain analysis (Figure 6A) showed that, on day 2 after infection, only KNZ01 remained in the subcutaneous tissues (dyed purple), whereas ATCC 12394 and KNZ03 were eliminated. Nevertheless, H&E-stained pathological images showed no significant differences among the three SDSE strains (Figure 6B). These results indicated that KNZ01 could remain in the subcutaneous tissues after infection, which resulted in a significant decrease in body weight of mice and severe erythema on day 2 after infection.

### Biofilm Formation of the KNZ01 Strain in the Presence of Human Serum

The cell surface structures of the SDSE strains were observed via SEM. To examine whether the cell surface structures are affected by human-derived source (serum), we compared THY medium-treated cells and those treated with RPMI 1640 supplemented with 10% fresh human serum (RPMI/hSerum).

**FIGURE 7:**
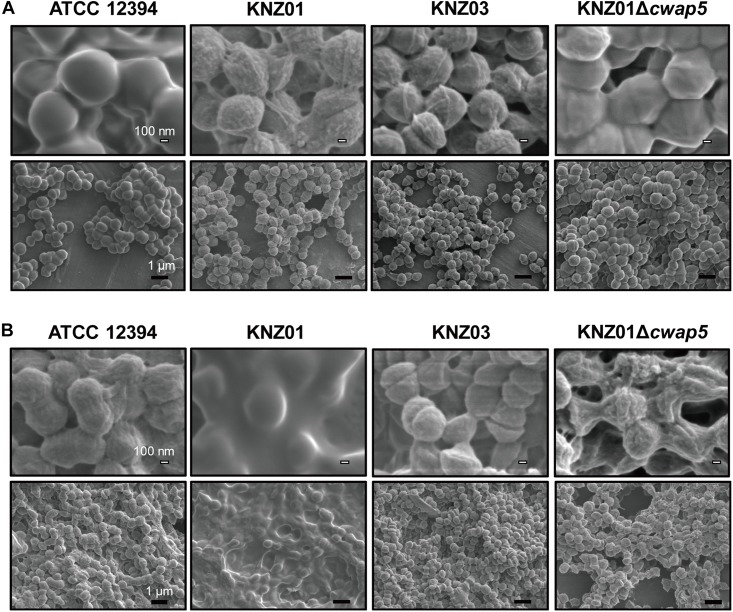
Scanning Electron Microscopy analysis of SDSE strains cultured in human serum-containing medium. SEM images of SDSE strains incubated in **(A)** THY medium or **(B)** RPMI 1640 containing 10% human serum. SDSE strains were cultured overnight in THY medium or RPMI 1640 supplemented with 10% fresh human serum at 37°C. Bacterial cells were fixed with 2.5% glutaraldehyde and washed twice with PBS. After serial dehydration with 50, 80, and 100% ethanol, the samples were soaked in 100% *t*-butyl alcohol, freeze-dried, and coated with 60% Au-Pd alloy. SEM images were obtained using JSM-7100F. White and black bars indicate 100 nm and 1 μm, respectively.

After overnight culture in THY medium, ATCC 12394 exhibited a smooth cell surface structure, whereas KNZ01 and KNZ03 had rough surfaces (Figure 7A, top); however, the cell surface of ATCC 12394 was rough after overnight culture in RPMI/hSerum (Figure 7B, top); also, KNZ01 constructed a biofilm with a large number of extracellular substances after overnight culture in RPMI/hSerum. These results showed that ATCC 12394 and KNZ01 construct different cell surface structures in response to culture conditions. KNZ01 specifically formed significant biofilms in the presence of human serum. Additionally, we found that the biofilm formation of KNZ01 partially decreased by the deletion of a gene encoding a cell wall-anchoring protein (*cwap5* gene in Supplementary Table S2), which indicated that the biofilm was partially composed of cell wall-anchoring proteins. No significant change was observed in the KNZ03 between THY medium and RPMI/hSerum, suggesting that KNZ03 lacks the response of the construction of cell surface structures to culture conditions.

## Discussion

In this study, we investigated the characteristics of SDSE such as genomic features, hemolytic activity, adherence to keratinocytes, anti-phagocytic activity, growth in the presence of serum, biofilm formation, and *in vivo* skin infection using the three strains (KNZ01, KNZ03, and ATCC 12394), which are categorized into prevalent *emm* and MLST groups in Japan and other countries (Wajima et al., 2016).

Among the three strains, KNZ03 exhibited the highest hemolytic activity to sheep RBC, while most of the activity was suppressed by trypan blue, an SLS inhibitor (Taketo and Taketo, 1982; Figure 2A). Gene analysis showed that the three strains had similar SagA, SagB, and SagC sequences, which form an SLS complex. Contrariwise, a lysine (Lys97) in the SLO of CC25 strains, including ATCC 12394 and KNZ03, was substituted for asparagine in those of the CC17 strains including KNZ01 (Supplementary Figure S1A). Both recombinant CC25 and CC17 SLOs exhibited significant hemolytic activity to sheep RBCs, while the activity of CC25 SLO was slightly higher than that of CC17 SLO (Supplementary Figure S1B). Uchiyama et al. (2015) reported that SLO influences bacterial anti-phagocytosis via the rapid impairment of neutrophil oxidative burst and antibacterial responses. As shown in Figure 4A, KNZ03 exhibited the highest survival rate after opsonization with the human serum and 30 min incubation with human neutrophils. However, Western blot analysis showed that SLO was not observed in the culture supernatant of KNZ03 (Figures 2B,C). SLO is also known to exhibit cytotoxic activity against mammalian cells (Bhakdi et al., 1985; Palmer, 2001). As shown in Figure 2D and consistent with the Western blot data, KNZ03 did not induce significant cell death to human keratinocytes (HaCaT cells). These results indicated that KNZ03 releases SLS for hemolysis and escapes from neutrophils through a non-SLO-mediated mechanism. The amount of SLO in KNZ03 was not changed by the protease inhibitor cocktail (Figure 2B), suggesting that KNZ03 SLO was not digested by some of its proteases but expressed only a few. Although previous reports show that SLO expression was regulated with the two promoters (Savic et al., 2002; Zhu et al., 2015), neither mutation or deletion was found in the promoter sequences of KNZ03 genome. It remains unknown how SLO expression is repressed in KNZ03.

Streptococci possess various virulence factors, such as fibronectin-binding protein, laminin-binding protein, collagen-binding protein, M protein, plasmin receptor, and pilus for adherence to host cell surface, as listed in Supplementary Table S1. Among the three strains, KNZ01 and KNZ03 adhered more significantly to human keratinocytes (HaCaT cells), compared with ATCC 12394 (Figure 3A); however, the difference in adherence between KNZ01 and KNZ03 was not significant (*P* = 0.16). We found that only KNZ01 possessed one gene that encoded a putative collagen-binding protein (Supplementary Table S1, yellow) using the Virulence Factors Database webtool (Chen et al., 2004). A BLAST search revealed that the putative protein is conserved in a part of SDSE (UniProtKB, ID C5WIP9) and *Staphylcoccus aureus* (UniProtKB, ID A0A380EV31) strains. A collagen-binding protein of *Streptococcus mutans* is involved in not only cell attachment, but also, in the hemorrhagic stroke of mice (Nakano et al., 2011). Besides, DFAST webtool showed that KNZ01 and KNZ03 possess a gene that encodes another collagen-binding protein (UniprotKB, A0A1F0CBD7) although this protein is also annotated as a cell wall-anchoring protein (cell wall-anchoring protein 8 in Supplementary Table S2). These results suggested that these collagen-binding proteins might be involved in the adherence ability of KNZ01 and KNZ03 to the human cell surface.

Biofilms are complex multicellular communities surrounded by a mature matrix of extracellular DNA, proteins, and polysaccharides that link bacterial cells (Stoodley et al., 2002; Marks et al., 2014). About 90% of GAS biofilms are mainly composed of hyaluronate, and the formation is involved in anti-phagocytic activity and antibiotic resistance (Baldassarri et al., 2006; Lembke et al., 2006; Ma et al., 2017). As SDSE lacks *hasA* and *hasB* genes, it does not produce hyaluronate. Nevertheless, recent reports showed that SDSE can produce biofilms. Genteluci et al. (2015) reported that 59.3% of 118 group C human SDSE isolates possessed the potential to form biofilms both *in vitro* and *in vivo*. Also, Ma et al. (2017) reported that 46.7% of 246 group G SDSE isolates showed moderate or strong biofilm-forming ability *in vitro*. Here, we found that the three strains exhibited different biofilm formation activities via SEM analysis (Figure 7). In response to a culture medium with high glucose (4.5 mg/ml) and 10% human serum, KNZ01 produced significant biofilms, whereas KNZ03 did not (Figure 7B). Glucose concentration regulates biofilm-associated genes of *S. mutans* (Shemesh et al., 2007). Besides, serum affects the biofilm formation of bacteria. Human serum inhibits the biofilm formation of *Pseudomonas aeruginosa* and *Candida albicans* (Hammond et al., 2010; Ding et al., 2014). Contrastingly, Yin et al. (2017) reported that serum from burn-injured mice promoted the biofilm formation in *S. aureus*. However, it remains unclear what induces the SDSE strain KNZ01 biofilm formation. Ma et al. (2017) proposed that ancillary proteins and sortases are related to biofilm formation; cell wall-anchoring proteins containing LPXTG motifs are designated on cell walls by sortases and are involved in the biofilm formation of SDSE. O’Neill et al. (2009) reported that biofilm phenotypes of *S. aureus* are mediated by the LPXTG-anchored fibronectin-binding proteins. The possession of *cwap* genes, which encode putative cell wall-anchoring proteins, varied among the three SDSE strains (Supplementary Table S2), indicating that the variety of these proteins are related to biofilm formation in the absence or presence of glucose and serum. Here, we found that KNZ01 biofilm was decreased by deletion of the *cwap5* gene, which encodes an LPXTG cell wall anchor domain-containing protein conserved in *Streptococcus agalactiae* (Figure 7B), suggesting that the cell wall-anchoring protein might be involved in the construction of SDSE biofilms. Additionally, biofilms generally adhere to surfaces of glass and polystyrene. Based on these biofilm formation data, we also examined whether the SDSE biofilms are involved in adherence to polystyrene surfaces (Supplementary Figure S2). After incubation with THY medium, KNZ03 bound to the polystyrene surfaces at a significantly higher rate than ATCC 12394 and KNZ01, although the appearance of biofilm obtained by SEM analysis showed no significant difference among the three strains. Conversely, after incubation with human serum (RPMI/hSerum), KNZ01 bound significantly to polystyrene surfaces but KNZ03 did not; the deletion of the *cwap5* gene did not affect the binding rate. These results indicated that the biofilm formation of the SDSE strains is not directly involved in their adherence to polystyrene surfaces.

Besides *in vitro* experiments, we examined subcutaneous infection in mice (Figure 5A). All the three SDSE strains induced ulcers on day 7 after infection, and the sizes of the ulcers did not differ significantly. Nevertheless, KNZ01 infection resulted in a significant body weight loss in mice (Figure 5B). Consistent with the body weight loss, on day 2 after infection, only KNZ01 remained in the mice skin tissues (Figure 6A), indicating that KNZ01 remained at the infection site for a longer time and induced more damages than the other two strains. SDSE exhibits pathogenicity to elderly people, especially those with underlying diseases such as tumorigenesis and diabetes mellitus. In the present study, we did not utilize any disease model of mice. We previously reported that intraperitoneal injection of SDSE to diabetic mice resulted in the abundant release of inflammatory cytokines, chemokines, and damages associated with molecular patterns, whereas injection into wild-type mice did not (Ogura et al., 2018); this suggested that pathogenicity of SDSE varies depending on the underlying diseases of the host. To elucidate SDSE pathogenicity, further analysis using disease model mice is required.

In conclusion, we analyzed pathogenicity of one CC17 strain (KNZ01; *stG*6792) and two CC25 strains (KNZ03; *stG*245 and ATCC 12394; *stG*166). *In vitro* and *in vivo* data indicated that KNZ01 exhibits pathogenicity to mice by possessing several virulence factor genes not conserved in the other two strains. Although both KNZ03 and ATCC 12394 are categorized into an identical CC and showed similar pathogenicity to mice skin, characteristics of their virulence factors, such as hemolytic activity, cytotoxicity, and anti-phagocytic activity were quite different. Our study proposed that the SDSE strains possess different characteristics in producing virulence factors for pathogenicity to humans.

## Data Availability Statement

Publicly available datasets were analyzed in this study. This data can be found here: NCBI BIOSAMPLE accession numbers – SAMN12206521 and SAMN12206522.

## Ethics Statement

The animal study was reviewed and approved by the Ethical Committee of Kanazawa University.

## Author Contributions

MM performed the experiments, analyzed the data, and drafted the manuscript. KO directed the study and revised the manuscript critically for important intellectual content. HS conducted the SEM analysis. TM-A conducted the data collection of whole-genome sequencing. YT-S collected the clinical isolates. YI contributed to genomic analysis. TW and SO conceived the study and contributed to the launch of this project. All authors improved the quality of the manuscript.

## Conflict of Interest

The authors declare that the research was conducted in the absence of any commercial or financial relationships that could be construed as a potential conflict of interest.

## Footnotes

1 http://www.atcc.org/products/all/12394.aspx

2 http://www.cdc.gov
