# Supplementary material for: Pathogenicity Characterization of Prevalent-Type Streptococcus dysgalactiae subsp. equisimilis Strains
Source: Front Microbiol. 2020 Feb 4;11:97. doi: 10.3389/fmicb.2020.00097 (PMC7010647; doi:10.3389/fmicb.2020.00097)
Supplement: Supplementary file 1 [file Data_Sheet_1.PDF]

Supplementary Material

Pathogenicity characterization of prevalent-type *Streptococcus dysgalactiae* subsp. *equisimilis* strains

Miki Matsue, Kohei Ogura*, Hironori Sugiyama, Tohru Miyoshi-Akiyama, Yukiko Takemori-Sakai, Yasunori Iwata, Takashi Wada, Shigefumi Okamoto

*** Correspondence:** Kohei Ogura: ogura@staff.kanazawa-u.ac.jp

Supplementary Methods

Hemolysis assay using recombinant SLOs

CC17 and CC25 *slo* genes were amplified using KNZ01 and KNZ03 genomes as templates, respectively. The utilized primers of the *slo* genes and pCold I plasmid (Takara Bio, Japan) are listed below. The genes were subcloned into the pCold I plasmid with Gibson Assembly Master Mix (New England Biolabs, E2611). The SLO proteins were obtained from BL21(DE3) cells transformed with the constructs and purified with Ni-NTA agarose (QIAGEN, 30210) according to the manufactures' manuals, followed by buffer exchange with PBS using PD-10 columns (GE Healthcare, 17085101). Protein concentration was measured by BCA Protein Assay Kit (Thermo Scientific, 23227). The sheep RBC solution were incubated in the presence of 50 mM 2-mercaptoethanol with the indicated concentrations of CC17 or CC25 SLO for 1 h at 37°C. After centrifugation at 2000 × g for 5 min, the absorbance of the supernatants was measured at a wavelength of 540 nm.

Primer-*slo*-Fwd, 5'CGAAGGTAGGATGAAGGACATGTCTAATAAAAAAATATTTAAAAAATAC

Primer-*slo*-Rvs, AGATTACCTACTACTTATAAGTAATCGAACCATATG

Primer-pCold-Fwd, TTATAAGTAGTAGGTAATCTCTGCTTAAAAG

Primer-pCold-Rvs, TGTCCTTCATCCTACCTTCGATATGATG

Biofilm assay

The capacity of SDSE strains to form biofilms on polystyrene surfaces was tested using a microtiter plate. The SDSE strains were grown overnight in THY medium, centrifuged, and resuspended in fresh THY medium or RPMI 1640 supplemented with 10% fresh human serum at OD_{600} = 0.01. The 200 µL of bacterial solutions were inoculated into the wells of a Nunc™ Microwell™ 96-well polystyrene microplate (Thermo Fisher Scientific, MA, USA) and incubated at 37ºC for 20 h under a 5% CO_2 atmosphere. The biofilms formed were washed with dH_2O to remove non-adherent cells,

dried at 70ºC, and stained with 0.1% crystal violet for 30 min. Excess dye was removed, and biofilms were washed with dH_2O and dried completely. After adding 300 μL of dimethyl sulfoxide (FUJIFILM Wako Pure Chemical Corporation) to the biofilms and incubation for 20 min at room temperature, the 200 μL of supernatants were transferred to a new microplate and absorbance at OD_{595} was measured.

Supplementary Figure Legends

Figure S1. Hemolytic activity of recombinant SLOs.

(A) Sequence Alignment of SLOs using CLUSTALW. (B) Hemolytic activity. 5% suspension of sheep RBC was incubated with the indicated concentrations of CC17 or CC25 SLO in the presence of 50 mM 2-ME (activator of SLO) for 1 h at 37°C. After centrifugation, the absorbance of supernatants was measured at 540 nm. Data are mean ± standard deviation of three (10 ng/ml and 2 ng/ml) or two (5 ng/ml and 1 ng/ml) separate triplicate experiments. *P* values were calculated by Student's T-test. "ns" means not significant.

Figure S2. The capacity of the SDSE strains to form biofilms on polystyrene surfaces.

The OD_{595} of the biofilms formed by the SDSE strains cultured in (A) THY medium or (B) RPMI 1640 supplemented with 10% fresh human serum (RPMI/hSerum). The SDSE strains were cultured in THY medium or RPMI/hSerum in a 96-well polystyrene microplate at 37ºC for 20 h. After washing with dH_2O, the biofilms were stained with 0.1% crystal violet for 30 min. The stained biofilms were treated with dimethyl sulfoxide for 20 min and the absorbance of the supernatants was measured at 595 nm. Data are mean ± standard deviation of two separate (n=6) experiments. $^{**}P < 0.01$, calculated by one-way analysis of variance (ANOVA) with Tukey–Kramer multiple comparison test. "ns" means not significant.

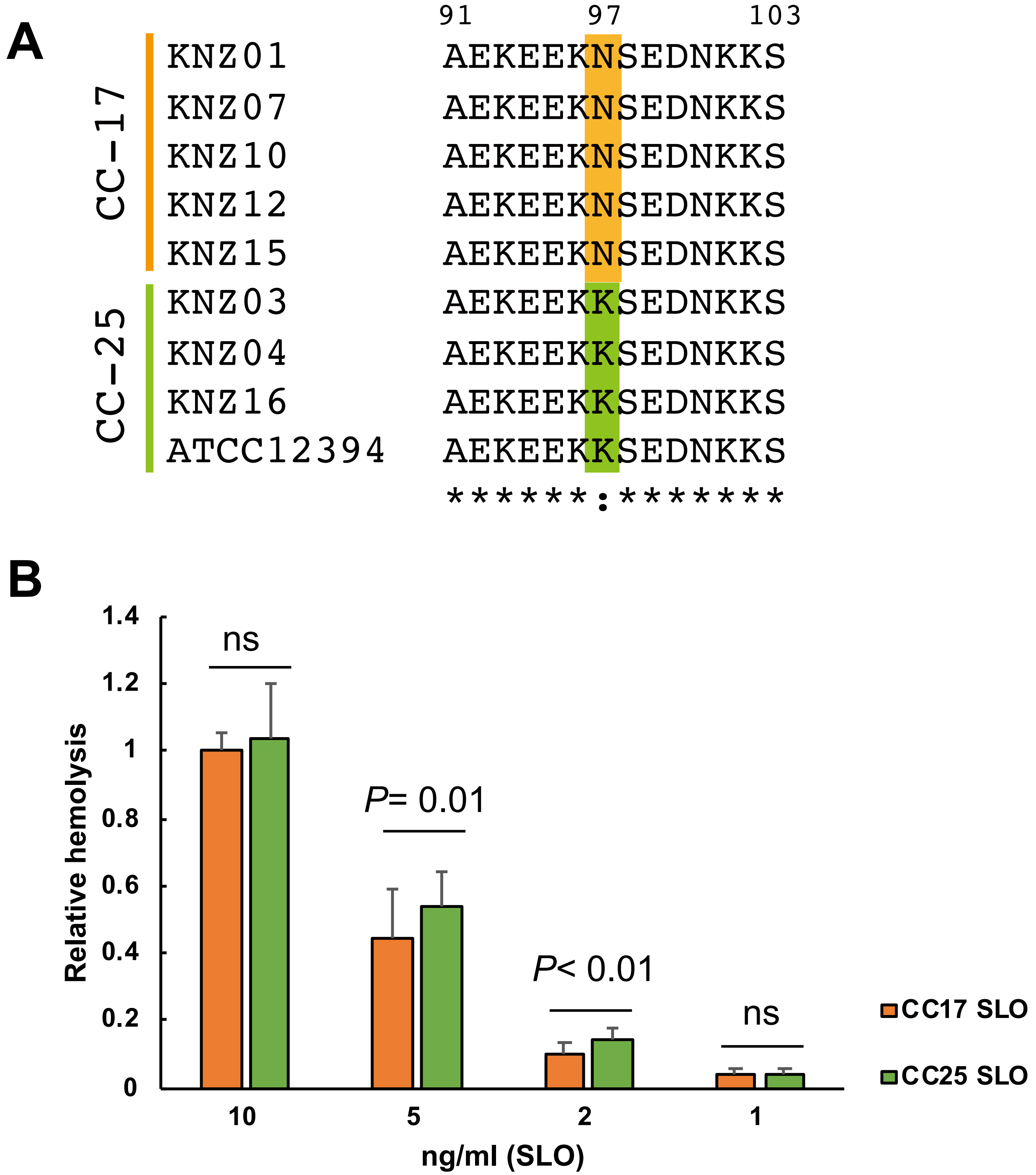
A
91
97
103
CC−17
KNZ01 AEKEEKNSEDNKKS
KNZ07 AEKEEKNSEDNKKS
KNZ10 AEKEEKNSEDNKKS
KNZ12 AEKEEKNSEDNKKS
KNZ15 AEKEEKNSEDNKKS
CC−25
KNZ03 AEKEEKKSEDNKKS
KNZ04 AEKEEKKSEDNKKS
KNZ16 AEKEEKKSEDNKKS
ATCC12394 AEKEEKKSEDNKKS
******:*******
B
Relative hemolysis
1.4
1.2
1
0.8
0.6
0.4
0.2
0
ns
P= 0.01
P< 0.01
ns
10
5
2
1
ng/ml (SLO)
CC17 SLO
CC25 SLO

Figure S1

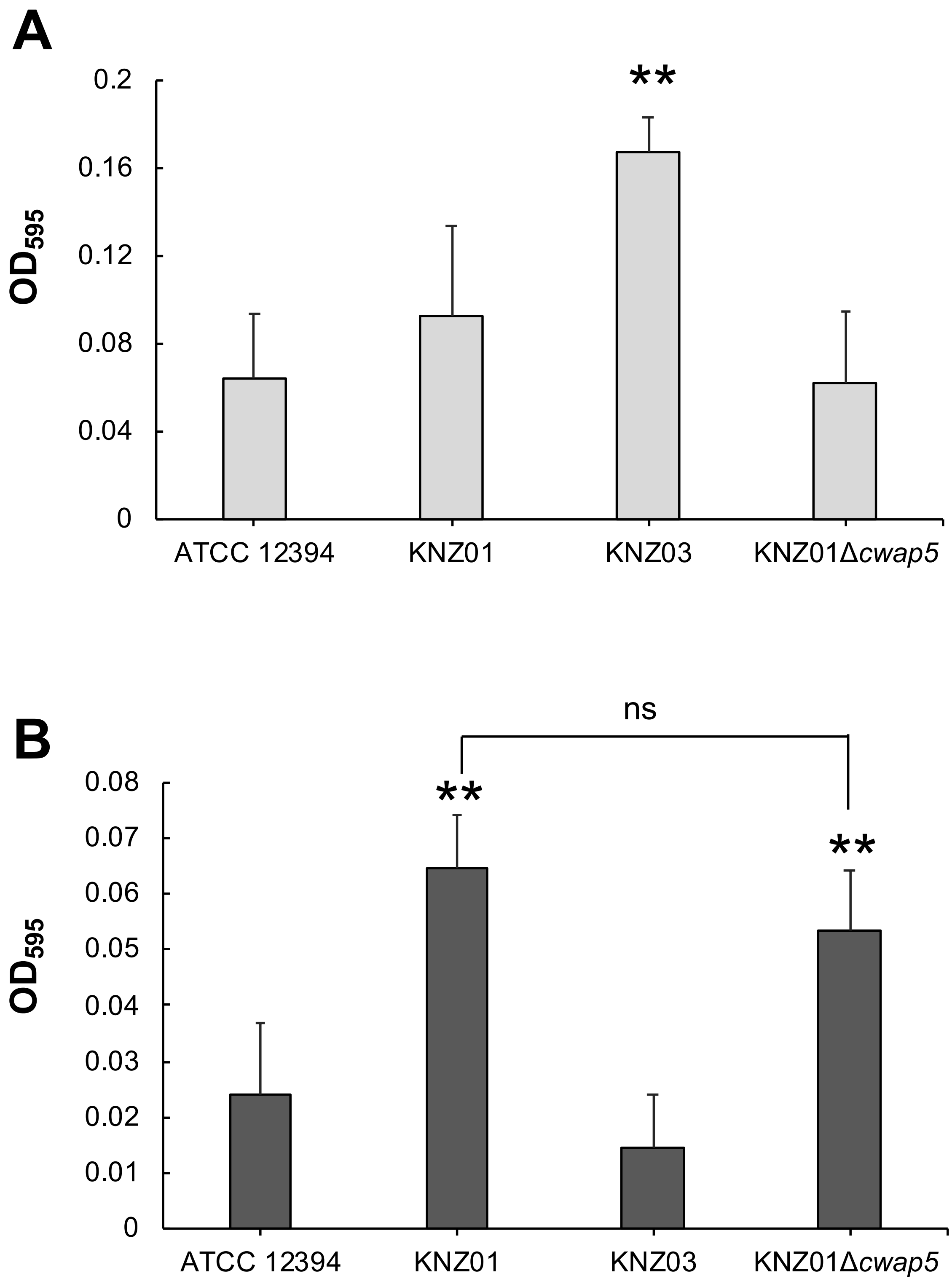
A
0.2
0.16
0.12
0.08
0.04
0
OD_{595}
**
ATCC 12394
KNZ01
KNZ03
KNZ01Δ*cwap5*
B
ns
0.08
0.07
0.06
0.05
0.04
0.03
0.02
0.01
0
OD_{595}
**
**
ATCC 12394
KNZ01
KNZ03
KNZ01Δ*cwap5*

Figure S2

Table S1. Virulence Factor Genes

VFclass	Virulence factors	Related genes	ATCC 12394 NC_017567.1	KNZ01 Draft genome	KNZ03 Draft genome
Adherence	Agglutinin receptor	*Undetermined*	+	-	-
	Antigen I/II (AgI/II) family of oral streptococcal adhesins	*sspA*	-	-	-
		sspB	-	-	-
	Antigen I/II (SpaP)	*spaP/pac*	-	-	-
	Cell surface hydrophobicity proteins	*cshA*	-	-	-
		cshB	-	-	-
	Choline-binding proteins	*cbpD*	-	-	-
		cbpG	-	-	-
		lytA	-	-	-
		lytB	-	-	-
		lytC	-	-	-
		pce/cbpE	-	-	-
		pspA	-	-	-
		pspC/cbpA	-	-	-
	Collagen binding protein	*cpa*	-	+	-
		cpbA	-	-	-
	Fibronectin-binding proteins	*fbaA*	-	-	-
		fbp54	+	+	+
		fbsA	-	-	-
		fbsB	-	-	-
		pavA	-	-	-
		prtF2	-	-	-
		prtF2	-	-	-
		sfbI/prtF1	-	-	-
		sfbII/sof	-	-	-
		sfbX	-	-	-
	GRAB	*grab*	-	-	-
	Glucan-binding proteins	*gbpA*	-	-	-
		gbpC	-	-	-
		gbpD	-	-	-
	Laminin-binding protein	*lmb*	+	+	+
	M protein	*emm*	+	+	+
	M-like proteins	*enn*	-	-	-
		mrp	-	-	-
	Muramidase-released protein	*mrp*	-	-	-
	Pilus island 1	*Undetermined*	+	+	+
		Undetermined	+	+	+
		Undetermined	+	+	+
		Undetermined	+	+	+

		Undetermined	-	+	+
	Pilus island 2	*pilA*	-	-	-
		pilB	-	-	-
		pilC	-	-	-
		srtC3	-	-	-
		srtC4	-	-	-
	R6 suface protein	*Undetermined*	-	-	-
	Serine-rich surface glycoproteins	*gspB*	-	-	-
		hsa	-	-	-
	Sortase A	*srtA*	-	-	-
	Streptococcal collagen-like proteins	*sclA*	-	-	-
		sclB	-	-	-
	Streptococcal glucosyltransferases	*gtfB*	-	-	-
		gtfC	-	-	-
		gtfD	-	-	-
		gtfG	-	-	-
	Streptococcal lipoprotein rotamase A	*slrA*	-	-	-
	Streptococcal plasmin receptor/GAPDH	*plr/gapA*	+	+	+
	Wall-associated protein A	*wapA*	-	-	-
	rlrA islet	*rrgA*	-	-	-
		rrgB	-	-	-
		rrgC	-	-	-
		srtB	-	-	-
		srtC	-	-	-
		srtD	-	-	-
Enzyme	EndoS	*endoS*	-	-	-
	Hyaluronidase	*hyl*	+	+	+
		hylA	-	-	-
		hylB	+	+	+
		hylP	-	-	-
		hysA	-	-	-
	Mitogenic factor 2	*mf2*	-	-	-
	Mitogenic factor 3	*mf3*	-	-	-
	Mitogenic factor 4	*mf4*	-	-	-
	Mitogenic factor	*mf/spd*	-	-	-
	Neuraminidase A	*nanA*	-	-	-
	Streptococcal enolase	*eno*	+	+	+
	Streptococcal phospholipase A2	*slaA*	-	-	-
	Streptodornase-alpha	*sda*	+	+	+
	Streptodornase	*sdn*	-	-	-
Immune evasion	Capsule	*Undetermined*	+	+	+
	SIC	*sic*	-	-	-

	Polysaccharide capsule(Bacillus)	*galE*	+	+	+
Immunoreactive antigen	Alpha C protein	*bca*	-	-	-
	Alpha-like protein	*alp2*	-	-	-
	Beta C protein	*cba*	-	-	-
	Rib	*rib*	-	-	-
	Surface immunogenic protein	*sip*	-	-	-
Iron uptake	Pneumococcal iron acquisition	*piaA*	-	-	-
	Pneumococcal iron uptake	*piuA*	-	-	-
Manganese uptake	Pneumococcal surface antigen A / Metal binding protein SloC	*psaA*	+	+	+
Protease	C3-degrading protease	*cppA*	*-	*-	*-
	C5a peptidase	*scpA/scpB*	+	+	+
	Extracellular factor	*epf*	-	-	-
	IdeS	*ideS/mac*	-	-	-
	IgA1 protease	*iga*	-	-	-
	Serine protease	*htrA/degP*	+	+	+
	SpeB/cysteine proteinase	*speB*	-	-	-
	Streptokinase A	*ska*	+	+	+
	Trigger factor	*tig/ropA*	+	+	+
	Zinc metalloproteinases	*zmpB*	-	-	-
		zmpC	-	-	-
Superantigen	Mitogenic exotoxin Z	*smeZ*	-	-	-
	Streptococcal pyrogenic exotoxin A	*speA*	-	-	-
	Streptococcal pyrogenic exotoxin C	*speC*	-	-	-
	Streptococcal pyrogenic exotoxin G	*speG*	-	-	-
	Streptococcal pyrogenic exotoxin H	*speH*	-	-	-
	Streptococcal pyrogenic exotoxin I	*speI*	-	-	-
	Streptococcal pyrogenic exotoxin J	*speJ*	-	-	-
	Streptococcal pyrogenic exotoxin K	*speK*	-	-	-
	Streptococcal pyrogenic exotoxin L	*speL*	-	-	-
	Streptococcal pyrogenic exotoxin M	*speM*	-	-	-
	Streptococcal superantigen	*ssa*	-	-	-
Toxin	ADP-ribosyltransferase SpyA	*spyA*	-	-	-
	Beta-hemolysin/cytolysin	*acpC*	-	-	-
		cylA	-	-	-

		cylB	-	-	-
		cylD	-	-	-
		cylE	-	-	-
		cylF	-	-	-
		cylG	-	-	-
		cylI	-	-	-
		cylJ	-	-	-
		cylK	-	-	-
		cylX	-	-	-
		cylZ	-	-	-
	CAMP factor	*cfa/cfb*	-	-	-
	Pneumolysin	*ply*	-	-	-
	Streptolysin O	*slo*	+	+	+
	Streptolysin S	*sagA*	+	+	+
	Suilysin	*sly*	-	-	-
Phagosome arresting	Nucleoside diphosphate kinase(*Mycobacterium*)	*ndk*	+	+	+

*Although the strains possessed cppA genes, encoded C3-degrading proteases lack catalytic cysteines.

Table S2. Putative cell wall surface anchor protein genes

					Result of protein BLAST search		
gene	size of gene (bp)	ATCC 12394	KNZ01	KNZ03	Aligned protein	source	NCBI accession ID
cell wall surface anchor protein 1	1695	+	-	-	isopeptide-forming domain-containing fimbrial protein	*S. dysgalactiae*	WP_014611941
cell wall surface anchor protein 2	1458	+	-	-	LPXTG cell wall anchor domain-containing protein	*S. dysgalactiae*	WP_014612201.1
cell wall surface anchor protein 3	1881	-	+	-	isopeptide-forming domain-containing fimbrial protein	*S. dysgalactiae*	WP_042357813.1
cell wall surface anchor protein 4	2670	-	+	+	VWA domain-containing protein	*S. dysgalactiae*	WP_015016563.1
cell wall surface anchor protein 5	2823	-	+	-	LPXTG cell wall anchor domain-containing protein	*S. agalactiae*	WP_001088038.1
cell wall surface anchor protein 6	762	-	-	+	LPXTG cell wall anchor domain-containing protein	*S. dysgalactiae*	WP_065354930.1
cell wall surface anchor protein 7	1743	-	-	+	isopeptide-forming domain-containing fimbrial protein	*S. dysgalactiae*	WP_143876923
cell wall surface anchor protein 8	924	-	+	+	LPXTG cell wall anchor domain-containing protein	*S. dysgalactiae*	WP_143876924.1
					Collagen binding protein	*S. agalactiae*	WP_000815034.1
